# Time-varying exposure and incomplete target-trial emulation in the dose–response study of early mobilization after cardiac surgery: a methodological commentary

**DOI:** 10.1186/s40560-026-00931-1

**Published:** 2026-08-19

**Authors:** Amolakhji, Pehlaj kumar

**Affiliations:** Liaquat University of Medical and Health Sciences Jamshoro, Sindh, Pakistan

To the Editor,

We read with interest Hirakawa and colleagues’ retrospective cohort study examining the dose–response relationship between early out-of-bed mobilization and functional decline among 610 patients requiring ≥3 days of ICU care after cardiovascular surgery [1]. Using 1:1 propensity-score matching on 196 patient pairs, the authors report that a mobilization dose of <2 units/day (each unit = 20 min of out-of-bed activity at IMS ≥ 4) was associated with a higher incidence of ≥5-point Barthel Index decrease at discharge (31.6% vs. 19.9%; OR 2.03; 95% CI 1.19–3.45) and, in a complementary continuous analysis, with a lower BI change score (β −5.38; 95% CI − 9.14 to − 1.62) [1]. We respectfully raise four methodological points that, taken together, suggest these findings should be interpreted as exploratory and hypothesis-generating and that future work should adopt more rigorous designs.

A 5-point decrease in the BI is designated “a clinically meaningful deterioration in functional independence,” yet the published minimal clinically important difference of the BI is highly population-specific: Hsieh and colleagues demonstrated that a mean change of only 1.85 points already exceeds measurement error and is perceived by stroke patients as important [2]. No analogous MCID has been derived for postcardiac surgical patients, and the BI’s step sizes of 0/5/10/15 mean that a 5-point swing can cross either a meaningful or a chance threshold depending on the item affected. The STROBE statement requires that outcomes be clearly defined using criteria appropriate to the population studied; a population-derived MCID obtained through an accepted anchor- or distribution-based approach would substantially reduce outcome misclassification, particularly for patients near the floor or ceiling of the 0–100 range [3].

The authors themselves acknowledge that “mobilization dose accumulated over time during ICU stay and was influenced by patients' evolving clinical status…should be interpreted as a time-varying exposure rather than a baseline intervention assigned at a single time point” [1]. The primary analysis nonetheless matches only on baseline covariates (age, sex, BMI, preoperative BI, APACHE II, duration of mechanical ventilation, and postoperative days to standing) and compares the average ICU exposure between groups—a snapshot precisely unsuited to a continuously updated intervention [1]. The reported PS model achieved a C-statistic of only 0.68 (95% CI 0.63–0.72) [1], and Haukoos and Lewis emphasize that “residual confounding may still exist if unmeasured factors influenced treatment selection” [4]. Simulation work specifically framed around critical-care severity adjustment shows that lower-accuracy risk adjustors (AUROC < 0.66) cause beneficial treatments to be falsely labeled harmful, with measured odds ratios for mortality of 1.4 or higher produced when the true beneficial effect was an OR of 0.6–0.8 [5]. The authors should, therefore, report *E* values or quantitative bias analyses for unmeasured confounding, alongside checks of post-matching residual imbalance in time-varying clinical covariates, such as new-onset atrial fibrillation, evolving shock, and rescue ventilation.

The paper states that the analysis was “conceptually framed as an emulation of a hypothetical target trial” [1]. The published framework, however, demands explicit specification of eligibility criteria, treatment strategies, assignment procedures, follow-up start, and outcome ascertainment, and recommends g-methods such as inverse-probability-weighted marginal structural models for interventions whose intensity is updated over time and which involve treatment-affected time-varying confounders [6]. The current design collapses the continuously updated intervention into a single between-group contrast using baseline-only PS, and this design cannot fully account for reverse causality, temporal alignment between exposure and outcome, treatment-affected time-varying confounding, or potential immortal-time bias [1, 6]. It is important to note, however, that the authors' sensitivity analysis restricting exposure to the first 72 h after ICU admission was designed to mitigate reverse causality and to improve temporal alignment between the exposure window and the outcome; it was not intended to address the *structural* immortal-time bias that arises in the primary contrast—a bias that operates independently, because patients must survive long enough in the ICU to accrue “high-dose” exposure and is not eliminated simply by narrowing the analytic window [1, 6]. The two concerns are, therefore, distinct, and readers should not interpret the existing 72 h sensitivity analysis as already resolving the guarantee-time/immortal-time issue we raise here. Because the original study was a retrospective single-center cohort that did not claim to provide definitive causal evidence, the most balanced interpretation is that the causal implications of the reported associations should be drawn cautiously, and that future prospective studies should consider formal target-trial emulation or marginal structural models to more rigorously handle treatment-affected time-varying confounding [6].

Patients who died in the ICU or were transferred early postoperatively were excluded from the primary analysis, preferentially retaining the most clinically stable patients; the worst-case sensitivity analysis reclassifies them as having experienced functional decline (OR 2.32; 95% CI 1.60–3.35) [1]. Mortality, postoperative transfer, and functional decline are, however, qualitatively distinct outcomes, and reclassifying death as functional decline conflates two different events. Because the primary outcome here is functional decline at discharge rather than a time-to-event endpoint, Fine–Gray or cause-specific hazards modeling would require redefinition of the outcome and follow-up structure and is not directly applicable to the present analysis; a more productive direction would be for future prospective work to adopt hierarchical or composite endpoints that jointly incorporate mortality, transfer status, and functional decline, so that each event type is captured without artificial reclassification [1]. With respect to analytic precision, STROBE item 10 calls for transparent reporting of how the analytical sample was reached and how the study size was determined [3]; the present study reports neither an a priori sample-size estimation nor a post hoc power assessment, despite a matched cohort of only 196 per group and multiple subgroup and restricted-cubic-spline analyses. We emphasize that, given the exploratory design, the absence of such a calculation should be read as a marker that the reported associations are hypothesis-generating rather than confirmatory—a reminder that confirmatory inference awaits adequately powered prospective replication—rather than as a reason to dismiss the findings themselves [1].

## Implications

Considered together, our comments are intended to position the findings of Hirakawa and colleagues’ study as exploratory associations that warrant cautious interpretation. Anchoring the primary outcome to a population-derived MCID, treating ICU death and early postoperative transfer as distinct from functional decline through hierarchical or composite outcome definitions in future prospective work, conducting adequately powered confirmatory replication, and applying formal target-trial emulation or marginal structural models for genuinely time-varying interventions in subsequent studies would help this patient cohort move from hypothesis-generating signal to more defensible evidence on individualized early-mobilization strategies after prolonged ICU stay.

## Data Availability

No datasets were generated or analyzed during the current study.
